# Flexible, Stretchable, and Self-Healing MXene-Based Conductive Hydrogels for Human Health Monitoring

**DOI:** 10.3390/polym17192683

**Published:** 2025-10-03

**Authors:** Ruirui Li, Sijia Chang, Jiaheng Bi, Haotian Guo, Jianya Yi, Chengqun Chu

**Affiliations:** 1 State Key Laboratory of Extreme Environment Optoelectronic Dynamic Measurement Technology and Instrument, North University of China, Taiyuan 030051, China; csj15035900480@163.com (S.C.); bjh18940966090@163.com (J.B.); sz202306095@st.nuc.edu.cn (H.G.); chuchengqun@nuc.edu.cn (C.C.); 2 School of Mechatronic Engineering, North University of China, Taiyuan 030051, China; 20180134@nuc.edu.cn

**Keywords:** hydrogel, MXene, self-healing, hydrogel-based sensors

## Abstract

Conductive hydrogels (CHs) have attracted significant attention in the fields of flexible electronics, human–machine interaction, and electronic skin (e-skin) due to their self-adhesiveness, environmental stability, and multi-stimuli responsiveness. However, integrating these diverse functionalities into a single conductive hydrogel system remains a challenge. In this study, polyvinyl alcohol (PVA) and polyacrylamide (PAM) were used as the dual-network matrix, lithium chloride and MXene were added, and a simple immersion strategy was adopted to synthesize a multifunctional MXene-based conductive hydrogel in a glycerol/water (1:1) binary solvent system. A subsequent investigation was then conducted on the hydrogel. The prepared PVA/PAM/LiCl/MXene hydrogel exhibits excellent tensile properties (~1700%), high electrical conductivity (1.6 S/m), and good self-healing ability. Furthermore, it possesses multimodal sensing performance, including humidity sensitivity (sensitivity of −1.09/% RH), temperature responsiveness (heating sensitivity of 2.2 and cooling sensitivity of 1.5), and fast pressure response/recovery times (220 ms/230 ms). In addition, the hydrogel has successfully achieved real-time monitoring of human joint movements (elbow and knee bending) and physiological signals (pulse, breathing), as well as enabled monitoring of spatial pressure distribution via a 3 × 3 sensor array. The performance and versatility of this hydrogel make it a promising candidate for next-generation flexible sensors, which can be applied in the fields of human health monitoring, electronic skin, and human–machine interaction.

## 1. Introduction

The performance of flexible sensors, as core components in the fields of human–computer interaction [1,2], health monitoring [3], electronic skin (E-skin) [4,5], and motion detection [6,7], directly determines the system’s sensing accuracy and response speed to external stimuli (e.g., pressure, strain, temperature, humidity). The flexibility and excellent stretchability of wearable electronic devices are crucial to achieve conformal attachment to irregular biological tissues, which can avoid discomfort and interface failures caused by mechanical mismatch between electronic devices and biological tissues. Although elastomer- and nanofiber-based electronics demonstrate exceptional conductivity and sensitive response characteristics, they are fundamentally constrained by inherent limitations, including restricted stretchability, interfacial incompatibility, non-biodegradability, and fabrication complexity, which severely impede their practical application translation. Notably, conductive hydrogels (CHs) have emerged as an ideal solution to overcome existing technological bottlenecks, owing to their intrinsic high extensibility (>1000% strain), superior compliance, and biocompatibility advantages [8]. However, at present, enabling conductive hydrogels to simultaneously possess ultrahigh stretchability, excellent electrical conductivity, and fast, sensitive biological signal sensing capabilities remains a major challenge [9,10,11]. This is because in conductive hydrogel composites, the enhancement of electrical conductivity and mechanical properties is often mutually constrained by the reduction in flexibility and deformability, making it difficult to achieve a balance between them [12,13]. Therefore, the development of hydrogel sensors capable of multimodal biological signal sensing while integrating excellent mechanical and electrical properties is of crucial importance, as it can greatly promote the advancement of human intelligence perception technologies.

The conductivity of hydrogels is typically achieved by incorporating conductive materials into a three-dimensional (3D) crosslinked polymer network-structured hydrogel matrix [14,15]. Researchers have developed CHs with superior electrical properties using various conductive materials such as carbon nanotubes [16], graphene [17], and MXene [18,19]. Among these, MXene, an emerging class of two-dimensional transition metal carbides/carbonitrides, has become a highly promising alternative material due to its exceptional metallic conductivity, large specific surface area, excellent hydrophilicity [14], and high ion intercalation capacity [20], leading to its widespread application in sensors and related technologies [21]. As the most representative type of MXene material, the band structure of Ti_3_C_2_T_x_ exhibits greater chemical tunability compared to other two-dimensional materials (such as molybdenum disulfide, tin selenide, and black phosphorus) [22]. The abundant surface functional groups (-O, -OH) of Ti_3_C_2_T_x_ and its negatively charged hydrophilic nature endow it with excellent water dispersibility, and it can form strong interactions with polymer networks through hydrogen bonding and electrostatic interactions, thereby enhancing the mechanical properties of hydrogels [23]. More importantly, Ti_3_C_2_T_x_ also possesses excellent electrical conductivity comparable to that of graphene, which can help form continuous conductive pathways inside the hydrogel, thus achieving high electrical conductivity [24]. Under strain or pressure, the high aspect ratio of Ti_3_C_2_T_x_ nanosheets allows them to act as “nanoscale reinforcing fillers” in the hydrogel network structure. These nanosheets can bridge polymer chains, dissipate energy under stress, and thereby improve the toughness of the hydrogel. Meanwhile, the sliding and restacking of Ti_3_C_2_T_x_ nanosheets in the hydrogel matrix significantly change the number and length of conductive pathways, leading to changes in resistance [25]. This property makes such hydrogels highly promising for applications in flexible electronic devices [26].

For the preparation of a 3D crosslinked polymer network-structured hydrogel matrix, most current studies adopt double crosslinked hydrogels rather than simpler single-crosslinked hydrogels [27]. Single chemically crosslinked hydrogels (e.g., pure PAM gels) usually have high stretchability but poor mechanical toughness and resistance to fatigue fracture, as their covalent crosslinks are prone to irreversible breakage under repeated stretching or pressure [28]. In contrast, single physically crosslinked hydrogels (e.g., pure freeze–thawed PVA gels) rely on non-covalent interactions (such as hydrogen bonds and crystallite formation) [29]. Although they have better self-healing potential, they exhibit low mechanical strength and unstable mechanical properties under environmental disturbances. The double crosslinked network combines the advantages of both systems: the covalent crosslinks of PAM provide structural integrity to prevent excessive deformation, while the physical crosslinks formed by freeze–thawed PVA act as “dynamic sacrificial bonds”—they can break and re-form under stress to achieve energy dissipation, thereby improving the toughness of the hydrogel [30]. This mechanical synergy is crucial for wearable sensors, which need to withstand repeated deformation without structural failure. In addition, single physically crosslinked hydrogels are highly sensitive to water content; water loss or low-temperature environments can disrupt their physical crosslinking structure, leading to material brittleness and decreased conductivity. Although single chemically crosslinked hydrogels have better water stability, they lack efficient dynamic interactions and thus struggle to maintain conductivity under strain. The double crosslinked network mitigates these issues: the covalent crosslinks of the chemically crosslinked hydrogel ensure long-term structural stability of the material against water evaporation, while the crosslinks of the physically crosslinked hydrogel form a secondary “water-retention network” by forming hydrogen bonds with water molecules. This double-network structure can maintain stable electrical conductivity even under cyclic strain or humidity changes, which is essential for reliable real-time monitoring of physiological signals [31].

In this work, a multifunctional hydrogel sensor that combines high sensitivity, excellent stretchability, and self-healing property is proposed. The sensor utilizes polyvinyl alcohol (PVA) and polyacrylamide (PAM) as the hydrogel matrix materials, with hygroscopic lithium chloride (LiCl) and MXene (PVA/PAM/LiCl/MXene) working synergistically as the humidity-responsive and resistive sensing components. The Li^+^ cations form hydrogen bonds with the negatively charged surface of MXene and interact with its abundant functional groups. This modification strategy significantly enhances both the conductivity and strain/pressure sensitivity of the MXene-based hydrogel while fully preserving its stretchability, which is of critical importance for developing multifunctional wearable devices with outstanding sensing capabilities.

## 2. Materials and Methodology

### 2.1. Materials

Titanium aluminum carbide (Ti_3_AlC_2_, 200 mesh) was purchased from Jilin 11 Technology Co., Ltd. (Changchun, China) Lithium chloride (LiCl, 99%), potassium persulfate (KPS), and polyvinyl alcohol (PVA, 1799) were obtained from Shanghai Aladdin Biochemical Technology Co., Ltd. (Shanghai, China) Acrylamide (AM), N,N′-methylenebisacrylamide (MBAA), and N,N,N’-tetramethylethylenediamine (TEMED, 99%) were purchased from Macklin Biochemical Co., Ltd. (Shanghai, China).

### 2.2. Preparation of MXene

First, 1 g of LiF was dissolved in 20 mL of HCl solution. Then, 1 g of Ti_3_AlC_2_ powder was slowly added to the solution, followed by stirring at 35 °C for 24 h. The resulting mixture was repeatedly centrifuged with deionized water (3500 rpm, 5 min) until the pH of the supernatant exceeded 6. Subsequently, the precipitate was subjected to ultrasonic exfoliation for 60 min. Finally, the reaction solution was centrifuged at 3500 rpm for 1 h, and the upper dispersion was collected and freeze-dried to obtain MXene.

### 2.3. Preparation of PVA/PAM/LiCl/MXene Hydrogel

First, 8 g of PVA particles was added to 92 mL of deionized water and stirred at 80 °C for 5 h. Then, 4 g of acrylamide (AM), 100 µL of MBAA, 1.25 mL of potassium persulfate (KPS), and 0.5 g of LiCl were added into the solution and magnetically stirred for 1 h. After cooling, 30 µL of TEMED was added as a crosslinking agent under an ice bath to accelerate polymerization. The solution was then transferred to a mold and subjected to three freeze–thaw cycles to form the gel. Finally, the PAM/PVA/LiCl hydrogel was immersed in a glycerol and water (1:1) mixed solution for approximately 12 h to ensure complete solvent exchange. Afterward, the hydrogel was immersed in deionized water for dialysis to remove unreacted substance. The PAM/PVA/LiCl hydrogel was prepared. The preparation of PVA/PAM/LiCl/MXene hydrogel was achieved by adding the MXene with different mass fractions into the mixture solution.

### 2.4. Characterization

The cross-sectional morphology of the hydrogel was observed using a scanning electron microscope (SEM) (JSM-7200F, JEOL, Tokyo, Japan). In addition, the structure and composition of the hydrogel were further characterized using Fourier transform infrared (FTIR) spectroscopy (Bruker Tensor27, Bremen, Germany).

### 2.5. Mechanical Property Tests

Mechanical tests were conducted at room temperature using a SANSCMT6104 universal testing machine (Xinsansi Enterprise Development Co., Ltd., Shanghai, China). Uniaxial tensile tests were performed on rectangular samples (10.0 mm wide, 1.0 mm thick, and 30.0 mm long). The samples were fixed between two clamps at a specific initial distance and then stretched at a constant speed of 30 mm/min until failure occurred. Toughness was determined by integrating the area under the stress–strain curve. The elastic modulus was calculated from the stress–strain curve at a 500% elongation.

### 2.6. Multimodal Sensing Tests

To develop a hydrogel-based flexible sensor for detecting human motion and array sensors, wires were placed on the surface and the backside of the hydrogel. Prior to all sensing tests (mechanical, temperature, humidity, and human application tests), the hydrogel sensor was equilibrated at room temperature (25 ± 2 °C) for at least 2 h. The hydrogel was then encapsulated with PDMS. The base and curing agent were mixed in a fixed ratio, thoroughly blended, and poured onto the hydrogel. The composite was then dried at 60 °C for 3 h.

In the temperature monitoring test, the hydrogel sensor was fixed onto a heating plate set to different programmed temperatures. Based on the resistance changes in the hydrogel at different temperatures, real-time electrical signals were recorded using an electrochemical workstation (CHI660E, Shanghai Chenhua Instruments Co., Ltd., Shanghai, China).

In the humidity monitoring test, the hydrogel samples were placed in a well-sealed humidity chamber, where the humidity was controlled with a CGS-MT optical integrated testing platform. Changes in relative resistance were recorded using an electrochemical workstation.

The sensing performance of the hydrogel under different strains was investigated using a flexible electronics tester (THE FT2000, Shanghai Mifang Electronic Technology Co., Ltd., Shanghai, China). The relative change in resistance was calculated using the following formula:

(1) $$\frac{∆R}{R_{0}}=\frac{R-R_{0}}{R_{0}}\times 100\%$$

where R is the resistance under strain, and R_0_ is the initial resistance (without strain).

The conductivity of the hydrogel was analyzed using an electrochemical workstation and calculated using the following formula:

(2) $$\sigma =\frac{L}{R\times S}$$

where L, R, and S represent the length, resistance, and cross-sectional area of the hydrogel, respectively.

For the sensitivity coefficient test, the sensitivity of the CHs is determined using the following formula:

(3) $$GF=\frac{(R-R_{0})/R_{0}}{\varepsilon }=\frac{∆R/R_{0}}{\varepsilon }$$

where GF is the sensitivity coefficient of the hydrogel, R_0_ is the original resistance, R is the resistance after applying strain, and ∆R/R_0_ represents the relative change in resistance, which is directly related to the applied strain ε.

### 2.7. Application Tests

The hydrogel was attached to the joints of the human body to detect its electrical signal response characteristics. Additionally, it was affixed to the wrist to monitor human pulse signals. Furthermore, the hydrogel can be constructed as a pressure array sensor for the real-time detection of pressure distribution within a given space.

## 3. Results and Discussion

### 3.1. Fabrication and Characterization of PVA/PAM/LiCl/MXene Hydrogel

**Figure 1:**
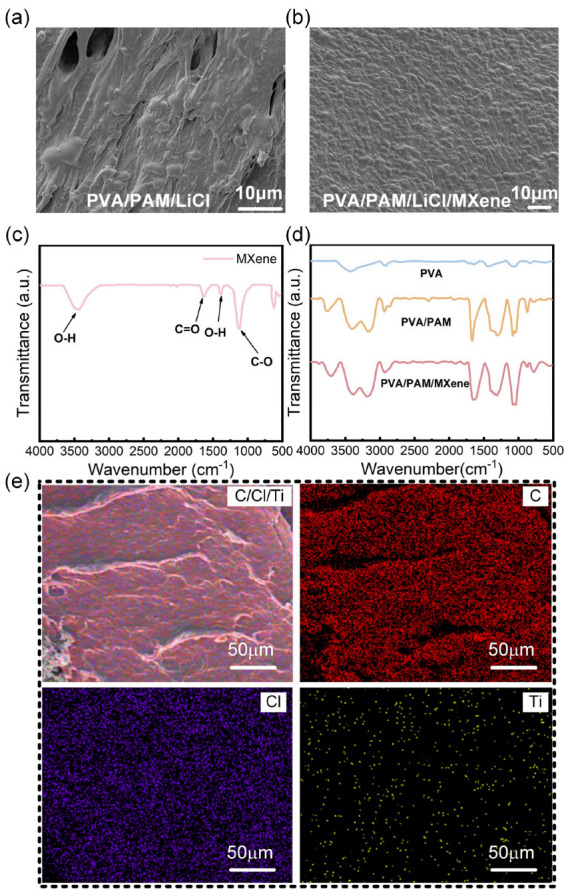
SEM images of (**a**) PVA/PAM/LiCl and (**b**) PVA/PAM/LiCl/MXene hydrogels. (**c**) FTIR pattern of MXene. (**d**) FTIR spectra of PVA, PVA/PAM, and PVA/PAM/MXene hydrogels. (**e**) EDS elemental mappings of PVA/PAM/LiCl/MXene hydrogel.

The PVA/PAM/LiCl and PVA/PAM/LiCl/MXene hydrogels were prepared separately, and their SEM images are shown in Figure 1a,b; the former has a certain pore structure, while the latter exhibits a dense network morphology. This morphological difference arises from synergistic interactions between MXene nanosheets and the PVA/PAM dual-network during gelation. MXene’s negatively charged surface (via deprotonated -OH/-O groups) forms hydrogen bonds with PVA’s -OH groups and electrostatic interactions with PAM’s polar amide groups, mediated by Li^+^ ions. These interactions act as “nanoscale crosslinking nodes,” restricting the random aggregation of polymer chains, guiding the formation of a compact network, and thus reducing pore size and increasing network density. The denser the network structure and the smaller the pore size, the more superior the mechanical properties of the hydrogel and the more favorable for energy dissipation [32,33,34]. The Fourier transform infrared (FTIR) results of MXene, PVA, PVA/PAM, and PVA/PAM/MXene are shown in Figure 1c,d. In Figure 1c, the infrared spectrum of the MXene nanosheets displays characteristic peaks at 3443 cm^−1^ (O-H), 1630 cm^−1^ (C=O), and 1119 cm^−1^ (C-O), which correspond to the abundant surface functional groups introduced during the preparation process [35,36]. In the PVA spectrum, the tensile vibrational absorption peak of O-H is shown at 3420 cm^−1^, with a broad peak shape due to the presence of hydrogen bonding conduction. The tensile vibrational absorption peak of O-H in-plane deformation and the tensile vibrational C-O absorption peaks are shown at 1430 cm^−1^ and 1070 cm^−1^, respectively [37]. However, based on the PVA spectra, the PVA/PAM and PVA/PAM/MXene spectra show distinct PAM characteristic peaks, such as the N-H symmetric and antisymmetric tensile vibrational absorption peaks of the primary amine group at 3400–3150 cm^−1^, the C-N tensile vibrational absorption peaks at 1050 cm^−1^, the C=O stretching vibration absorption peak at 1700 cm^−1^, and the C=C stretching vibration absorption peak near 1500 cm^−1^. When MXene is incorporated into the PVA/PAM matrix, the O-H stretching vibration peak of PVA (originally at 3420 cm^−1^) shifts slightly toward lower wavenumbers in the PVA/PAM/MXene hydrogel. This shift occurs because the -OH groups of PVA form hydrogen bonds with the -O/-OH groups on the MXene surface, which weakens the bond energy of the O-H bonds and consequently reduces their vibration frequency. Meanwhile, the symmetric/antisymmetric stretching vibration peaks of N-H (3400–3150 cm^−1^) and the stretching vibration peak of C-N (1050 cm^−1^) in PAM also exhibit slight broadening. This is attributed to the electrostatic interactions between the polar amide groups of PAM and the negatively charged surface of MXene, which induces minor changes in the local electronic environment of the functional groups in PAM. In addition, for PVA/PAM/MXene spectra, the C=O stretching peak at 1700 cm^−1^ broadens, confirming heterogeneous hydrogen bonding between MXene’s -OH and PAM’s C=O groups. This dense, small-pore morphology is not merely a structural observation. It directly dictates subsequent mechanical and conductive performance. Unlike the porous MXene-free hydrogel, in which large pores act as stress concentration points, the dense network of the MXene hydrogel enables uniform stress distribution and efficient ion transport. As illustrated in Figure 1e, to further analyze the distribution of MXene in the hydrogel, Energy-Dispersive Spectroscopy (EDS) elemental mapping analysis was conducted. The main elements of MXene are titanium (Ti) and carbon (C), while chlorine (Cl) originates from LiCl. The results show that Ti elements are uniformly distributed throughout the hydrogel matrix without obvious agglomeration, which strongly confirms the uniform dispersion of MXene in the polymer network.

### 3.2. Mechanical Properties and Self-Healing Performance

**Figure 2:**
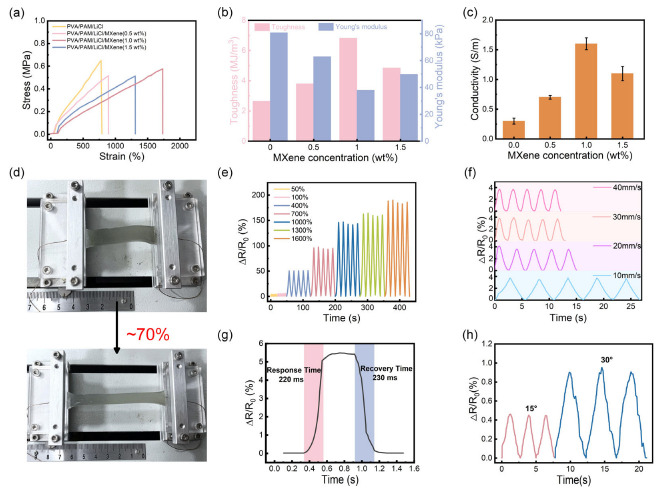
(**a**) Stress–strain curves of PVA/PAM/LiCl/MXene with different MXene concentrations and (**b**) the corresponding toughness and Young’s modulus. (**c**) Effect of MXene concentration on the conductivity of PVA/PAM/LiCl/MXene. (**d**) Physical image of the hydrogel under 70% strain. (**e**) Change curve of ∆R/R_0_ of PVA/PAM/LiCl/MXene hydrogel at different strains. (**f**) Strain curves at different stretching rates under 30% strain. (**g**) Response time and recovery time of the hydrogel under pressure. (**h**) Response at different bending angles.

The introduction of MXene into the PVA- and PAM-based double-network hydrogel further enhances the mechanical properties. Figure 2a,b show that after the addition of MXene, the breaking stress and elongation of PVA/PAM/LiCl/MXene hydrogel are significantly improved, the toughness is 2.5 times that of PVA/PAM/LiCl hydrogel, and Young’s modulus is 38 kPa (<60 kPa). As the MXene concentration increased to 1 wt%, the strain of the PVA/PAM/LiCl/MXene hydrogel increased to 1700%, which was much greater than that of PVA/PAM/LiCl (strain of 800%). This enhancement in stretchability is likely due to synergistic effect of the network regulation induced by MXene and the dual-crosslinked matrix (PVA physical crosslinking and PAM chemical crosslinking). Furthermore, the abundant functional groups (e.g., -OH, -O) on the MXene surface can form secondary crosslinking with the hydrogel matrix, enabling uniform dispersion of MXene in the polymer network [34]. Consequently, a dense polymer network is formed, which helps improve the toughness and stretchability of PVA/PAM/LiCl/MXene. Meanwhile, due to the unique metallic conductivity of MXene and the increased electron transfer in the 3D MXene nanoskeleton, incorporating MXene into the hydrogel matrix significantly enhances the electrical conductivity of PVA/PAM/LiCl/MXene. As shown in Figure 2c, when the MXene concentration is less than 1%, MXene nanosheets are too sparse to form sufficient crosslinking nodes with PVA/PAM, resulting in weak mechanical reinforcement and discontinuous conductive pathways (conductivity <1.0 S/m). The maximum electrical conductivity of the hydrogel reaches 1.6 S/m as the MXene content increases from 0 to 1 wt%. However, further increasing the MXene content from 1 wt% to 1.5 wt% causes the MXene nanosheets to aggregate (due to van der Waals forces between nanosheets). This disrupts the polymer network and creates “brittle zones” that reduce fracture strain (to ~1200%) and block ion transport. Table 1 compares the maximum strain of our hydrogel sensor with those of reported hydrogel sensors. Consequently, based on the mechanical and electrical properties mentioned above, 1 wt% MXene is considered the optimal concentration for further research.

To further investigate the potential of the PVA/PAM/LiCl/MXene hydrogel in wearable sensors, the strain sensitivity through dynamic stretching tests was examined. Figure 2d shows an optical photograph of the hydrogel being stretched using a fixture. As shown in Figure 2e, the hydrogel exhibited highly consistent and stable electrical resistance response characteristics at ambient temperature over a stretching range of 50–1600%. As shown in Figure 2f, a cyclic stretching test was conducted on the hydrogel with a fixed strain of 60%. The hydrogel exhibited good repeatability under different stretching rates, indicating stable cyclic performance at the same stretching rate. However, at lower stretching rates, the hydrogel’s response rate also slowed down, which demonstrates that the hydrogel’s stretching response has a certain dependence on rate. In addition, this hydrogel sensor can also respond to external pressure. By applying and removing pressure on the hydrogel, the pressure response curve of the hydrogel can be obtained, as shown in Figure 2g. Moreover, the hydrogel has a pressure response time of 220 ms and a recovery time of 230 ms, reflecting the fast response characteristic of the hydrogel sensor to external pressure. Subsequently, the hydrogel was fixed in a rotating fixture. By changing the bending angle, the corresponding relative resistance change value could be successfully observed, as shown in Figure 2h. This indicates that the hydrogel can be applied to stress detection at different bending interfaces, and in particular, it has certain application potential in human activity monitoring.

**Figure 3:**
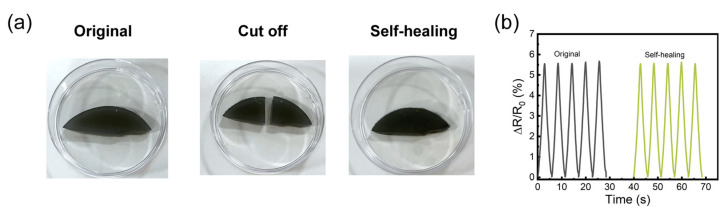
(**a**) Self-healing of PVA/PAM/LiCl/MXene hydrogel and (**b**) comparison of mechanical properties before and after self-healing.

In the PVA/PAM/LiCl/MXene hydrogel, MXene forms abundant hydrogen bonds and electrostatic interactions with the polymer matrix. These unique intermolecular forces significantly enhance the self-healing properties of the nanocomposite hydrogel. This self-healing ability can effectively extend the service life of the PVA/PAM/LiCl/MXene hydrogel in applications such as ionic skin and flexible sensors. Figure 3 characterizes the self-healing properties of the hydrogel. Specifically, as shown in Figure 3a, after the hydrogel sample was cut into two halves, the fractured surfaces were brought into contact with each other. After 4 h of natural healing, the cut surface was almost indistinguishable, indicating that the self-healing process is highly efficient and reliable. The self-healing property is driven by dynamic non-covalent interactions. In detail, the hydrogen bonds between MXene’s -OH/-O groups and PVA/PAM’s functional groups can reform at the fractured interface, and the Li^+^ ions (mobile in the hydrated matrix) re-establish electrostatic bridges between MXene and the polymer network. Moreover, the incorporation of glycerol not only imparts excellent water retention capability to the hydrogel but also markedly mitigates water loss during the healing process. This feature retains moisture to ensure the mobility of polymer chains, sustains the reversibility of the aforementioned interactions, and decelerates the decline in the internal ion migration rate, thereby effectively preserving the recovery of the hydrogel’s mechanical properties and electrical conductivity. To verify the self-healing recovery performance of the hydrogel, a repetitive stretching test was conducted at 100% strain for five cycles on both the original and the self-healed hydrogel. As shown in Figure 3b, the self-healed hydrogel showed mechanical properties almost identical to those of the initial hydrogel.

### 3.3. Temperature and Humidity Performance

**Figure 4:**
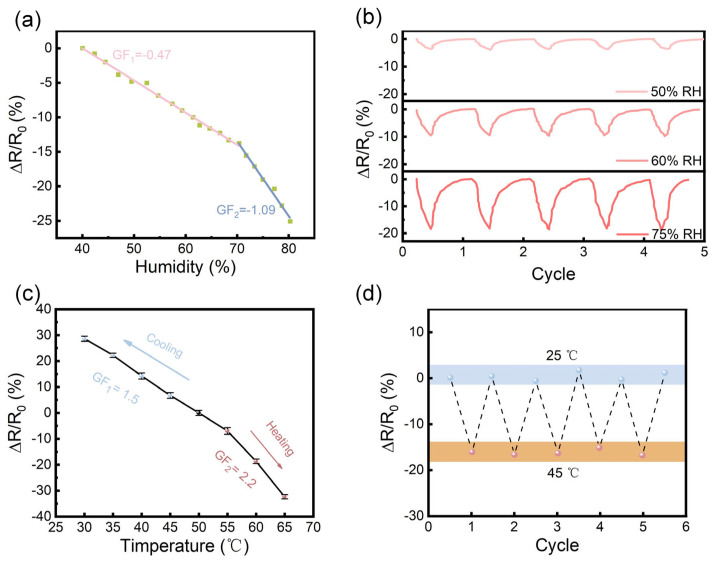
(**a**) Humidity sensitivity response curve of the PVA/PAM/LiCl/MXene hydrogel sensor. (**b**) Stability of the hydrogel’s relative resistance change at different humidity levels. (**c**) ∆R/R_0_ curve of hydrogel real-time response to temperature. (**d**) Excellent stability and repeatability after several cycles of hot and cold temperature variations.

MXene nanomaterials possess a large specific surface area, abundant surface functional groups, and excellent conductivity, making them highly responsive with broad humidity sensing potential. The adsorption/desorption of water molecules affects the interlayer spacing between MXene sheets. As water molecules are adsorbed onto the MXene surface, hydrogen bonds are formed between the water molecules and surface functional groups, gradually accumulating into a liquid water layer, which shows a response to humidity. Humidity sensitivity is a key performance indicator for evaluating humidity sensing capabilities. Figure 4a shows the resistance curve of the PVA/PAM/LiCl/MXene humidity sensor in relation to humidity. It can be observed that through linear fitting, the sensitivity curve of the humidity sensor can be divided into two linear regions (S_1_: 40–70% RH; S_2_: 70–80% RH). Particularly in the high humidity range of 70–80% RH, it exhibits a high response of −1.09, demonstrating high sensitivity and a good linear relationship. The response in 70–80% RH arises from MXene’s water adsorption behavior and Li^+^-mediated ion transport. Below 70% RH, water molecules are sparsely adsorbed on MXene’s surface, forming isolated hydrogen bonds with -OH groups, and only slightly increasing ion mobility. Above 70% RH, adsorbed water molecules accumulate into a continuous liquid layer, which dissociates LiCl into free Li^+^ ions. These Li^+^ ions (along with MXene’s metallic conductivity) create a highly conductive pathway, leading to a sharp resistance decrease. To further explore the reliability of its humidity sensing, the repeatability of relative resistance changes in cyclic hydration and dehydration tests was examined. As shown in Figure 4b, the hydrogel sensor showed stable and reversible humidity responses within the relative humidity range of 50% to 75%.

In addition to strain and humidity sensing, the PVA/PAM/LiCl/MXene hydrogel can also be used for temperature sensing. As shown in Figure 4c, the relative resistance decreases as the temperature increases. This is due to the increased mobility of charge carriers with the rise in temperature. As the temperature rises, polymer chain motion increases, reducing the resistance to Li^+^ ion migration and electron transfer across MXene nanosheets. With 55 °C as the reference point, the heating sensitivity (GF_1_) and cooling sensitivity (GF_2_) are 2.2 and 1.5, respectively. The higher heating GF than cooling GF reflects the asymmetry in charge carrier dynamics; specifically, heating rapidly enhances polymer chain motion, while cooling leads to the gradual rigidification of the chains. In addition, the PVA/PAM/LiCl/MXene hydrogel sensor maintains high repeatability and stability after several testing cycles. Figure 4d reveals significant differences in response to alternating hot and cold temperature changes. The PVA/PAM/LiCl/MXene hydrogel sensor is mounted on the wall of a beaker, into which hot and cold water are alternately added. When 45 °C hot water is added, the relative resistance rapidly decreases, and when 25 °C cold water is added, the relative resistance gradually increases, demonstrating the sensitivity of the temperature sensor. The temperature sensing characteristic indicates that the sensor has the potential to be applied to the real-time monitoring of human body temperature.

### 3.4. PVA/PAM/LiCl/MXene Sensor for Detection of Human Motion and Physiological Activities

**Figure 5:**
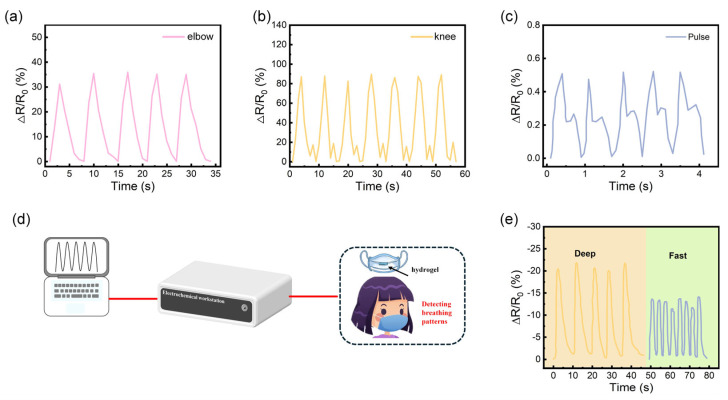
PVA/PAM/LiCl/MXene hydrogel as a strain wearable sensor for human motion detection: (**a**) elbow bending, (**b**) knee bending, (**c**) human pulse, (**d**) real-time monitoring of breathing rate. (e) humidity response curves for deep breathing and rapid breathing.

Based on the strong adhesion, fatigue resistance, and stable strain sensitivity of the PVA/PAM/LiCl/MXene ion-conductive hydrogel, this sensor, due to its excellent stretchability and ease of attachment to human skin, is applied as a wearable strain sensor for monitoring human motion signals. The hydrogel sensor can be directly adhered to body joints, including the elbow and knee, effectively and real-time converting various repetitive body movements into electrical signals. Different bending sites can be precisely recorded, and their relative changes can be distinguished based on the relative resistance curve (Figure 5a,b). For joint motion, the sensor’s ~1700% stretchability ensures it does not fracture during full elbow/knee bending, while its fast response time captures real-time motion signals. Moreover, the sensor can also detect subtle physiological characteristics, such as a pulse cycle of approximately 0.94 s, as shown in Figure 5c. The sensor’s ability to detect subtle physiological signals stems from its high strain sensitivity and conformal adhesion. When attached to the wrist, pulse-induced skin deformation alters the hydrogel’s conductive pathways, leading to measurable resistance changes. Therefore, these sensors, based on the PVA/PAM/LiCl/MXene hydrogel, can differentiate obvious and subtle strain signals caused by body movements or skin deformation, demonstrating great potential for monitoring human motion.

The developed MXene-based hydrogel exhibits high humidity sensitivity, making it suitable for respiratory monitoring to provide timely health information about the human body. The prepared sensor was attached inside a mask and connected to an electrochemical workstation as well as a computer, enabling the monitoring of breathing patterns, as shown in Figure 5d. When exhaled air from the mouth or nose passes through the PVA/PAM/LiCl/MXene hydrogel, the water vapor molecules it carries are captured and absorbed by MXene and Li^+^, which further stimulates the conductive network and causes changes in the electrical signal. The corresponding characteristics of the human body can be determined through different signal information such as amplitude, waveform, and frequency. As shown in Figure 5e, deep breathing allows the hydrogel to remain in a high-humidity state for a longer time, resulting in an extended signal peak. In contrast, rapid breathing shows a faster frequency in the curve, and the humidity is not as high as during deep breathing. Accurately detecting breathing patterns can expand the potential application of PVA/PAM/LiCl/MXene in the real-time monitoring of respiratory-related diseases, such as congenital asthma in infants.

**Figure 6:**
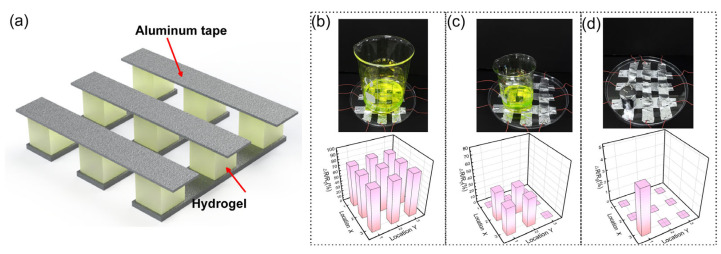
PVA/PAM/LiCl/MXene hydrogel assembled into pressure sensor matrix. (**a**) Schematic of 3 × 3 pressure sensor array. Schematics of the array placed in a beaker containing (**b**) 100 g of water, (**c**) 30 g of water, and (**d**) 100 g of weight placed on the array.

Furthermore, the mechanical sensing capability of the PVA/PAM/LiCl/MXene hydrogel sensor was investigated. A pressure-sensing array integrates multiple pressure-sensing units to detect spatial pressure signals over a large area and multiple points. The sensor array can not only sense pressure but also accurately locate the distribution of the pressure signals. This goal was accomplished by utilizing a 3 × 3 sensor array with a sandwich structure, allowing for the visualization of the planar pressure distribution, as shown in Figure 6, by recording the relative resistance changes in each individual unit in the matrix, and the pressure distribution can be reflected. As shown in Figure 6b–d, due to the uniform electrical conductivity and localized resistance variation characteristics of the hydrogel, the pressure array can clearly distinguish the precise location and intensity of the external pressure, laying the foundation for spatial pressure-sensing technology.

## 4. Conclusions

In this work, a multifunctional PVA/PAM/LiCl/MXene conductive hydrogel was successfully fabricated using a simple glycerol/water immersion strategy, leveraging PVA/PAM dual networks for structural support and MXene/LiCl for enhanced functionality. The mechanical and electrical properties of the hydrogel can be effectively enhanced through the presence of MXene and LiCl in the hydrogel network, and the stretchability also meets the basic requirements for wearable devices. At the optimized MXene concentration (1 wt%), the hydrogel exhibited exceptional comprehensive performance (~1700% elongation at break, a maximum electrical conductivity of 1.6 S/m, and efficient self-healing). The hydrogel exhibited reliable multimodal sensing capabilities, featuring high humidity sensitivity, excellent temperature responsiveness, and rapid pressure response/recovery times. Moreover, practical wearable applications have been further validated, including human joint movement detection, weak pulse signal detection, breathing pattern recognition, and distributed pressure measurement. This work provides a new insight for the application of hydrogel-based sensors in physiological signal monitoring within complex systems and holds great significance for the development of wearable electronic devices in the medical field. Furthermore, integrating this hydrogel with other functional modules (e.g., wireless transmission chips, energy harvesters) in the future will enable the development of self-powered portable sensing systems, further expanding its applications in remote health monitoring.

## Figures and Tables

**Table 1:** Maximum strain comparison between the PVA/PAM/LiCl/MXene hydrogel and reported MXene-based hydrogel.

Hydrogel System	MXene Loading	Maximum Strain	Ref.
MXene–sodium alginate–PAM-Ca^2+^/Li^+^ composite hydrogel	1 wt%	224.2%	[38]
PAM/sodium alginate/MXene double-network hydrogel	3.5 wt%	1400%	[32]
PAM/sodium alginate/MXene hydrogel	4 g	1500%	[39]
Dispersion-enhanced MXene hydrogel	0.76 wt%	800%	[40]
polyvinyl alcohol/polyacrylamide/MXene hydrogel	0.3 wt%	1033%	[41]
PVA/PAM/LiCl/MXene hydrogel	1 wt%	1700%	Our work

## Data Availability

The original contributions presented in this study are included in the article. Further inquiries can be directed to the corresponding author.
